# Ovarian Stromal Cell-Conditioned Media, but Not Co-Culture, Improves Survival in Feline Follicles

**DOI:** 10.3390/ani15111539

**Published:** 2025-05-24

**Authors:** Batsheva Marks, Jennifer Beth Nagashima, Carol L. Keefer, Nucharin Songsasen

**Affiliations:** 1 Department of Animal and Avian Sciences, University of Maryland, College Park, MD 20742, USA; batshevamarks98@gmail.com (B.M.); ckeefer@umd.edu (C.L.K.); 2 Center for Species Survival, Smithsonian’s National Zoo and Conservation Biology Institute, Front Royal, VA 22630, USA; nagashimaj@si.edu

**Keywords:** conditioned media, domestic cat, ovarian follicles, co-culture

## Abstract

Ovarian stromal cells play roles in in vivo folliculogenesis; however, little is known about their effect on in vitro cultured follicles. This study investigated the impact of ovarian stromal cell co-culture or conditioned medium (CM) on the survival and development of domestic cat follicles in vitro. Preantral (n = 148 follicles), early antral (n = 92), and antral (n = 22) stage cat follicles were divided into five groups (control, ovarian stromal cell co-culture, 20% CM, 50% CM, and 100% CM), cultured for 13 days, and evaluated for survival, growth, and the mRNA expression of *CYP19A*, *GDF9*, and *FSHR*. Additional follicles (n = 199) were isolated, divided into three groups (control, co-culture, and 100% CM), cultured for 10 days, and oocytes were subjected to in vitro maturation (IVM). More follicles (*p* ≤ 0.01) cultured in 100% CM survived until day 11 of culture than other groups. Antral follicle survival was significantly lower than pre- or early antral (*p* ≤ 0.0001). However, no differences (*p* > 0.05) in growth were detected across the treatments. *CYP19A* expression was upregulated (*p* ≤ 0.001) in the 50% CM-treated follicles. Furthermore, no differences (*p* > 0.05) were found in IVM rates between cultures. In summary, the findings demonstrate that conditioned medium collected from primary cultures of ovarian stromal cells improves in vitro survival of isolated cat follicles.

## Simple Summary

Preserving the genetic diversity of wildlife populations is a high priority for endangered species conservation efforts. One way to preserve genes from an animal that dies prematurely is rescuing immature gametes and cryopreserving or growing them in the lab. This study investigated the effect of co-incubating ovarian cells with ovarian follicles in the domestic cat (a model for endangered felids). We found that conditioned media collected from ovarian cell cultures significantly improved follicle survival, although there were no differences in the expression level of developmental markers between follicles cultured with conditioned media and the controls. Furthermore, oocyte maturation rates after culturing follicles were not significantly different among groups. Altogether, this study is an important step toward optimizing a laboratory system for maturing female gametes.

## 1. Introduction

In the mammalian ovary, thousands of primordial follicles are embedded within the cortex [1]. Through mechanisms that have not been fully characterized, primordial follicles activate and develop into primary, secondary, and antral stage follicles. However, only a few of these follicles will fully mature and ovulate a developmentally competent oocyte, leaving an untapped wealth of unused germplasm [1]. Replicating folliculogenesis in vitro is key to fertility preservation, which can be applied to endangered wildlife and women facing infertility associated with cancer treatments [2]. However, challenges to completing this process remain, especially in large, non-rodent models [3].

The domestic cat is a mid-sized mammalian model, which makes it ideal for human-focused ovarian research. It also serves as a model for endangered feline species [4]. To date, in vitro cultures of isolated early antral cat follicles in alginate hydrogel have resulted in the production of meiotically competent oocytes [5]; however, the current incubation conditions are far from optimal and do not support earlier-stage follicles [6]. Like other non-rodent models, there is a need to develop culture conditions supportive of follicle maturation that will ultimately result in the consistent production of developmentally competent oocytes.

One aspect limiting in vitro folliculogenesis success may be the lack of cross communication between isolated follicles and somatic cells present in the ovary, including ovarian stromal cells [7]. The ovarian stroma consists of extracellular matrix components (e.g., collagen and gelatin), endothelial cells, immune cells, ovary-specific components (e.g., tunica albuginea and ovarian surface epithelium), and fibroblast-shaped cells that have not been fully characterized [8]. Studies have shown that ovarian stroma cells regulate ovarian folliculogenesis in many ways. For example, blood vessels provide tissues with nutrients, hormones, and oxygen supplies while facilitating waste transport. In addition, ovarian stromal cells secrete paracrine factors, including bone morphogenetic protein-4 (BMP-4) and BMP-7 [9], both of which are known to regulate follicle growth. A recent study has shown that ovarian stromal cells maintain survival and promote the growth and estradiol production of isolated human preantral follicles [10]. Other cell types, including adipose-derived stem cells, mouse embryonic fibroblasts, theca cells, and endothelial cells, have also shown a beneficial impact on in vitro follicle development [11,12,13,14]. An alternative way to restore the missing paracrine signals, but without introducing a potentially confounding factor, such as cell overgrowth, is to incubate follicles in conditioned media obtained from cultured cell lines [15,16]. Culturing mouse preantral follicles in endometrial cell conditioned media improves preantral follicle development and increases the developmental competence of in vitro grown oocytes by maintaining granulosa cell viability and function [17]. Furthermore, conditioned media of human umbilical cord mesenchymal stem cells have been shown to improve follicle maturation in mice by stimulating the PI3K-Akt pathway [18].

To date, no research has been conducted to examine the impact of co-culture or conditioned media on in vitro follicle development in the domestic cat.

The objective of the present study was to determine the influence of ovarian stromal cell co-culture and conditioned medium supplemented at varying concentrations on feline in vitro folliculogenesis and the mRNA expression of three follicular health genes: *CYP19A*, *GDF9*, and *FSHR*. We hypothesized that the presence of ovarian stromal cells or conditioned medium would affect the growth and survival of preantral, early antral, and antral cat follicles.

## 2. Materials and Methods

All chemicals were obtained from Sigma Aldrich (St. Louis, MO, USA) unless otherwise stated.

### 2.1. Ovarian Stromal Cell Isolation and Culture

Cat ovarian stromal cells were enzymatically isolated using a modified method described previously [19]. Briefly, ovarian medullas (n = 3 cats) were finely minced, then placed in a digestive medium of phosphate-buffered saline (PBS), collagenase (0.5 mg/mL), and DNase I (0.01 mg/mL). The tissue was rocked at 37 °C for 30 min, then the medium was strained sequentially through a 100 μm filter and a 40 μm filter. The collected medium was transferred into a 15 mL conical tube (Fisher Scientific, Hampton, NH, USA) and an equal volume of PBS + 10% fetal bovine serum (FBS) was added to the tube. The tissue pieces were placed back in fresh digestive medium and incubated for an additional 30 min until the tissue sufficiently disassociated, then they were strained again. The collected medium and an equal volume of PBS + 10% FBS were added to the conical tube. The medium was centrifuged at 300× *g* for 5 min, then the pellet was washed with 1× RBC lysis buffer, followed by PBS + 1% bovine serum albumin (BSA), and then PBS + 0.04% BSA. Cells were cultured in an endothelial cell growth medium (EGM) (PromoCell, Heidelberg, Germany) + 10% FBS at 38 °C in humidified 5% CO_2_ until 70–80% confluent. Cells were passaged twice: first to a T25 flask, then to a T75 flask. After the second passage achieved confluency, cells were cryopreserved and stored in liquid nitrogen until used.

### 2.2. Cryopreservation, Recovery, and Co-Culture of Ovarian Stromal Cells

Cells (2nd passage) at a concentration of 2 × 10^6^ cells/mL were suspended in EGM supplemented with 10% FBS and 10% dimethyl sulfoxide. Suspended cells (1 mL) were placed into cryotubes (Fisher Scientific). The cryotubes were then placed in a Corning^®^ CoolCell^®^ container (Corning, NY, USA) and slowly cooled to −80 °C overnight. The next day, the cryotubes were plunged into liquid nitrogen for long-term storage. Cells were recovered by placing the tubes in a warm water bath at 37 °C for 2 min, then the contents were dropwise diluted with 9 mL EGM in a 15 mL conical tube. The tube was centrifuged at 300× *g* for 5 min, and the supernatant was discarded. The cell pellet was resuspended in EGM and cultured in a T25 flask at 38.5 °C in 5% CO_2_ until 80% confluent. At that point, the cells were passaged and cultured in EGM (-) (EGM + 2% FBS, without VEGF, IGF, and FGF supplementation). After 24–48 h, the conditioned medium (CM) was collected from the cell culture and stored at −80 °C until use. The cells were then passaged and seeded into a four-well dish (Fisher Scientific) at 10,000 cells/well for the co-culture treatment group. All co-culture cells were allowed to establish and grow for 24 h before follicular culture began. Remaining cells were cultured separately in a T75 until enough CM was produced for that given replicate, then discontinued.

### 2.3. Follicle Isolation and Encapsulation

Ovaries were collected from domestic cats older than 6 months. The ovaries were placed in a transportation medium (L-15 medium supplemented with 30 μg/mL penicillin G and streptomycin sulfate and 8.8 μg/mL ascorbic acid) and kept at 4 °C until processing. Ovaries with corpora lutea were discarded. All ovaries collected in a single day were pooled.

Follicular isolation was conducted within 6 h of surgery. All isolation procedures were performed in collection medium (minimum essential medium [MEM] supplemented with 2 mM L-glutamine, 50 U/mL penicillin G, 50 μg/mL streptomycin sulfate, 0.1 mg/mL ascorbic acid, and 3 mg/mL BSA) on 37 °C warming plates. The ovarian cortex was thinly sliced from the ovarian surface and washed in collection medium. Subsequently, follicles were micro-dissected from the surrounding cortex using a scalpel and 25-gauge needles under a dissection microscope (Nikon SMZ800N, Nikon Instrument Inc., Melville, NY, USA). The follicles were encapsulated in 0.5% alginate (*w*/*v*), as previously described [20]. Briefly, 2–3 follicles were washed in 100 μL alginate, then transferred to another drop of 100 μL alginate. Individual follicles were pulled up into a pipette, along with 4 μL of alginate, then dropped into a calcium salt solution (140 mM NaCl, 50 mM CaCl_2_) for 2 min to crosslink. Encapsulated follicles were transferred individually into each well of 24-well plates or pre-seeded 4-well co-culture plates containing 500 μL EGM (-), and supplemented with 1 μg/mL recombinant porcine FSH (Vetoquinol, Fort Worth, TX, USA). In each culture experiment (16 replicates performed on different days), follicles were distributed randomly across treatment groups, with a minimum of two follicles per treatment group.

On three collection days for the experimental cultures, fresh follicles of approximately 600–1000 μm were also isolated (n = 10 follicles), flash frozen, and stored at −80 °C for the subsequent qRT-PCR analysis.

### 2.4. Co-Culture and CM Treatment

Follicles (120 μm to 750 μm in initial diameter, n = 262 follicles, 40 cats) were collected and classified into three developmental stages based on their average initial diameter (ID): preantral ID ≤ 300 μm (n = 148); early antral 300 μm<ID≤500 μm (n = 92); and antral ID > 500 μm (n = 22). The follicles were randomly divided into five treatment groups: (1) EGM (-) (control, n = 52); (2) co-culture (EGM (-) + ovarian stromal cells; n = 51); (3) 20% CM, (80:20, EGM (-): CM; n = 53); (4) 50% CM (50:50, EGM (-): CM; n = 52); (5) 100% CM (100% CM; n = 54).

Follicles were observed and photographed on Days 0, 4, 6, 8, 11, and 13 using an EVOS microscope (FL Auto 2, Thermo Fisher, Waltham, MA, USA). Minimum and maximum follicle diameters were measured using Image J (v1.53t) [21]. The two diameters were then averaged. The following formula was used to calculate the percentage of growth each day:

$$Growth=\frac{Day\;Average\;Diameter-ID\;}{ID}\times 100$$

Positive growth was defined as greater than 5% average diameter expansion. Average diameter changes between −5% and 5% was determined to be static. Average diameter changes less than −5% for two consecutive time points indicated follicular death at the first time point.

All follicles were maintained in culture for 13 days, regardless of survival status. Co-culture cells were also photographed during follicle diameter assessment to monitor proliferation, morphology, and health. On day 13, follicles were removed from culture and flash frozen for qRT-PCR analysis.

### 2.5. RNA Isolation, cDNA Production, and qRT-PCR Analysis

RNA isolation was performed using an RNeasy kit (74004, Qiagen, Hilden, Germany). For RNA extraction, 100 μL of Buffer RLT was added to each frozen tube, and the follicles were homogenized using a mortar and pestle. Follicles from each treatment across multiple culture experiments were combined in a 1.5 mL Eppendorf tube to achieve 10–12 follicles per tube. This was defined as a technical replicate, with each replication containing follicles obtained from the same donor cats. Additional buffer RLT was then added to each Eppendorf tube to bring the final volume to 600 μL. The samples were homogenized again before proceeding according to the manufacturer’s instructions. There were five qRT-PCR technical replicates for all treatment groups, with the exception of the 20% CM and co-culture groups, which had only four replicates. This was due to a replicate’s low RNA concentration, which fell below the minimum required level for cDNA production.

cDNA was synthesized from 50 ng RNA using an iScript cDNA Synthesis Kit (1708890, BioRad, Hercules, CA, USA) according to the manufacturer’s instructions. Four genes were analyzed: *ACTG* (reference gene), *FSHR*, *CYP19A*, and *GDF9*. All reactions were performed in duplicate on a Roche LightCycler (Basel, Switzerland), with the manufacturer’s SYBR green I Master Mix (04707516001, Roche, Basel, Switzerland). The following settings were used for the qRT-PCR amplification: 300 s preincubation at 95 °C, 45 cycles amplification at 95 °C 15 s, 53 °C for 30 s, 72 °C for 30 s, and melting at 95 °C 10 s, 65 °C 60 s, and 97 °C 1 s. Primer sequences, optimal melting temperature, and product length are in Table 1. Standard curves were separately generated for the primers via serial dilutions of cDNA to analyze primer efficiency, as described by the Pfaffl method [22]. To obtain relative expression, the experimental treatments were normalized to the cultured control follicles.

### 2.6. Oocyte Maturation

To assess the development of in vitro-grown oocytes, additional follicles (n = 199 follicles, 26 cats, 11 replicates) were isolated and cultured for 10 days in one of the following conditions: control, co-culture, or 100% CM. At the end of the incubation, 30 μL of 30 μg/mL alginate lyase (A1603) was added to each culture well, and the follicles were incubated for 45 min at 38 °C until the alginate bead had dissolved. Follicles were collected and washed twice in follicle collection medium. Oocytes (n = 132) were released by breaking the follicle wall with a 25-gauge needle and washed once in oocyte maturation medium (Quinn’s Advantage™ Blastocyst Medium (ART-1029, Cooper Surgical, Trumbull, CT, USA), 5% FBS, 10 μg/mL FSH, 1 μg/mL LH). Oocytes were transferred to an oocyte maturation medium droplet under mineral oil, the volume of which was adjusted to 10 μL per oocyte. The oocytes were cultured for 24 h at 38 °C and 5% CO_2_. After culture, the oocytes were collected, washed twice in PBS, and placed in PBS + 0.2% sodium azide, 2% normal goat serum, 1% BSA, 0.1 M glycine, and 0.1% Triton X-100 [25]. The cumulus cells were removed by repeated pipetting. The oocytes were washed once more in PBS before being fixed in 4% paraformaldehyde for 24 h. Finally, the oocytes were stained with 10 μg/mL DAPI and mounted with ProLong Gold antifade reagent (2406594, Invitrogen, Waltham, MA, USA). The meiotic status of the oocyte was then classified as germinal vesicle (GV), germinal vesicle breakdown (GVBD), metaphase II (MII), or degenerated (D) [26].

### 2.7. Statistical Analysis

Statistical analysis was conducted with R Statistical Software (R version 4.3.2 (31 October 2023)) [27]. If a follicle demonstrated −5% to 5% static growth, it was normalized to 0% growth. If a follicle demonstrated negative growth for a timepoint without qualifying as dead, the follicle was included in that day’s average. Growth averages per day were then analyzed via a Kruskal–Wallis test with significance set at *p* ≤ 0.05. Pairwise comparisons were performed with a Wilcox test and a Benjamini–Hochberg *p*-value adjustment.

Survival was analyzed with respect to treatment and initial size with a Cox’s proportional hazards test, with the survival package in R and *p*-value significance at *p* ≤ 0.05 [28]. It was then plotted via the survminer package (v0.4.9) [29]. If significance between curves was found, the emmeans package (v.1.10.1) was used to test pairwise comparisons [30].

A chi-squared test was performed to determine any differences in oocyte maturation rates. Significance was set at *p* ≤ 0.05.

## 3. Results

### 3.1. Culture Conditions and Follicular Stage Effects on Follicle Survival and Growth

**Figure 1:**
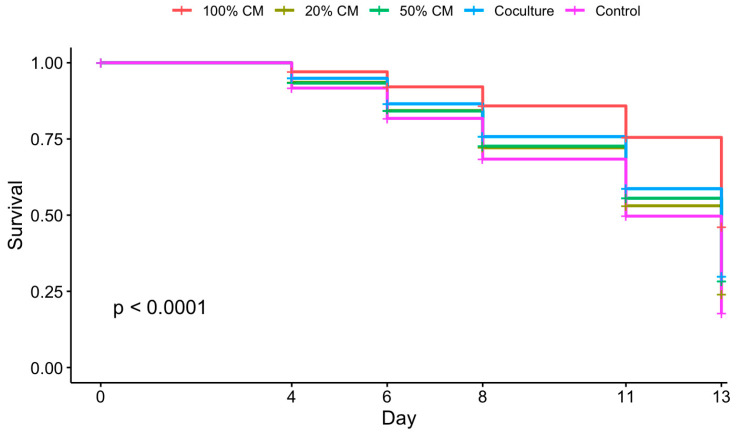
Survival curve of follicles based on treatment groups: 100% CM, 50% CM, 20% CM, co-culture, and control (n = 16 replicates, 262 total follicles). Cox proportional hazards test, *p* < 0.0001.

**Figure 2:**
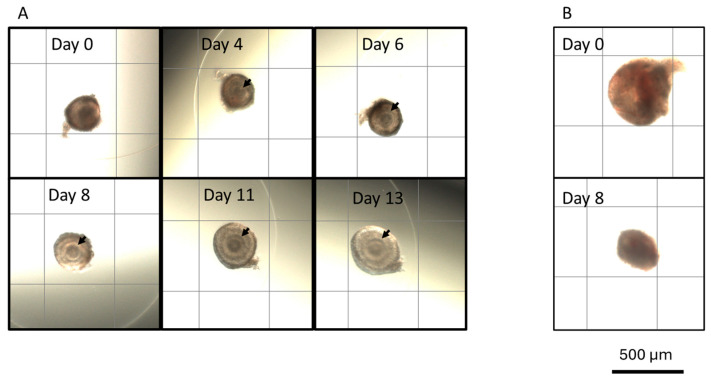
(**A**) Photographs of a cat ovarian follicle during 13-day in vitro culture. The oocyte is visible in the center of the follicle (arrows), especially after four days in culture. Total follicle growth was 45.8%. (**B**) Photographs of a non-viable cat follicle on days (D) 0 and 8 of in vitro culture. The follicle decreased in size and lacked a clearly defined basement membrane and oocyte after eight days in culture. Scale bar is 500 μm.

**Figure 3:**
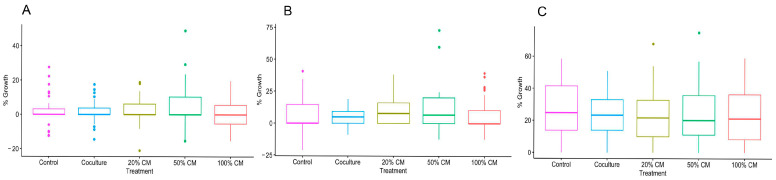
(**A**–**C**) box and whisker plot of percent growth of cat follicles on days (D) 4, 8, and 13, respectively, divided by treatment group (n = 16 replicates and 262 total follicles, *p* > 0.05).

To determine the impact of ovarian stromal cells and CM on in vitro folliculogenesis, follicles were cultured with cells or in medium supplemented with varying percentages of CM. Culture conditions influenced follicular survival (Figure 1, *p* < 0.05). Specifically, follicles incubated in 100% CM exhibited a higher survival rate than other culture groups. At day 13, the 100% CM group displayed a survival rate of 46%, compared to 17% in the control and 20–30% survival in other conditions (*p* ≤ 0.0001). The contrast was even more pronounced on days 8 and 11. Specifically, 86% and 75% of follicles incubated in 100% CM survived on days 8 and 11, respectively, but fewer than 76% did so in other groups on day 8, while 60% did so on day 11. However, culture conditions did not impact growth rates (*p* > 0.05) (Figure 2 and Figure 3).

**Figure 4:**
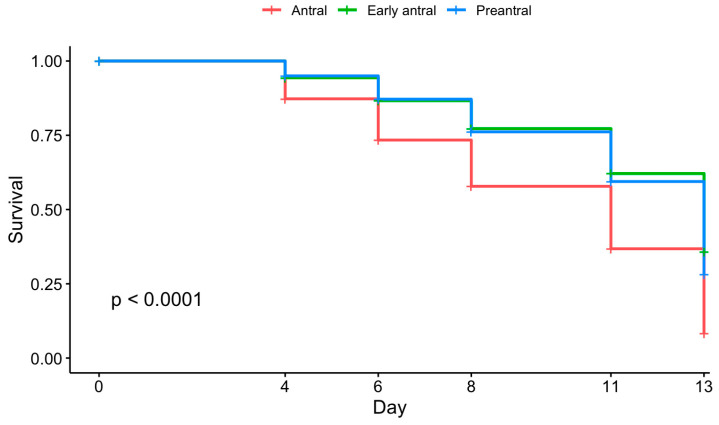
Survival curve by follicle initial size categorization of preantral, early antral, and antral (n = 16 replicates, 262 total follicles). Cox proportional hazards test, *p* < 0.0001.

Initial follicle size also impacted follicular survival (Figure 4, *p* ≤ 0.0001). Antral follicles displayed low survival rates at days 8 and 13, although there were no differences between preantral and early antral follicles. The survival rates at day 8 were 76%, 77%, and 58% for preantral, early antral, and antral follicles, respectively. By day 13, only 8% of antral follicles remained viable compared to 36% for early antral and 28% for preantral follicles.

**Figure 5:**
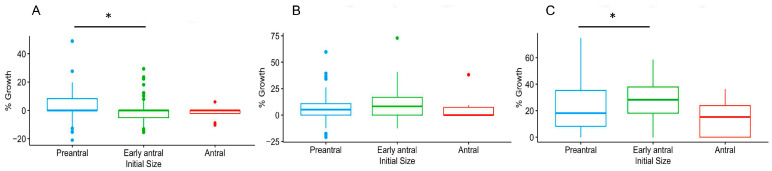
(**A**–**C**) box and whisker plot of percent growth on days (D) 4, 8, and 13, respectively, divided by initial size categories (n = 16 replicates and 262 total follicles, (*) *p* ≤ 0.05).

Initial follicle size also influenced growth rate (Figure 5, *p* ≤ 0.05). Growth rates were significantly different on day 4 and day 13 between preantral and early antral follicles. Specifically, the growth rate of preantral follicles was higher than the early antral stage on day 4. However, the opposite was observed on day 13. Only 40% of preantral follicles that survived to day 13 formed an antrum.

### 3.2. Impact of In Vitro Culture Conditions on Gene Expression

**Figure 6:**
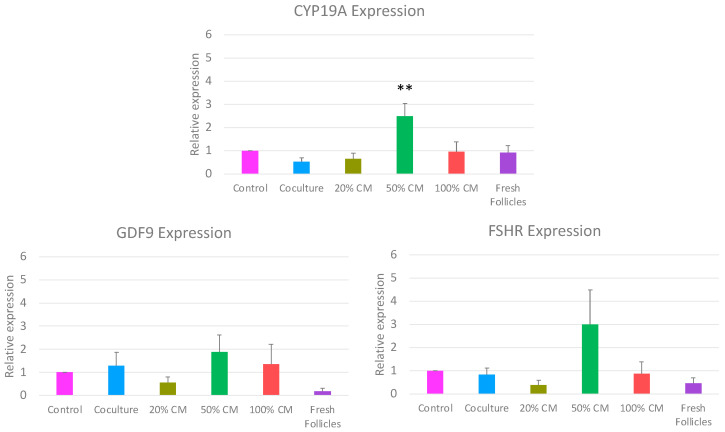
qRT-PCR Analysis of in vitro cultured follicles. Gene expression of *CYP19A*, *FHSR*, and *GDF9* of freshly isolated or 13-day in vitro cultured follicles (n = 5 replicates). Expression is relative to cultured control and normalized to *ACTG*, (**) *p* ≤ 0.001.

To identify if culture conditions impacted follicle function, the expression of three key follicular health markers was measured. In vitro culture conditions influenced the expression of *CYP19A*, but not *FSHR* and *GDF9* (Figure 6). Specifically, *CYP19A* expression increased about threefold in the 50% CM treatment group as compared to all other treatments (*p* ≤ 0.001).

### 3.3. Impact of In Vitro Culture Conditions on Oocyte Maturation

To determine if a meiotically mature oocyte could be obtained, follicles were cultured with ovarian stromal cells or CM for 10 days, and the resident oocytes were subjected to IVM. Culture conditions did not impact the developmental competence of recovered oocytes. There were no oocytes that achieved metaphase II after in vitro culture and maturation. Furthermore, there were no differences in rates of GV, GVBD, and degenerated oocytes (*p* > 0.05). Percentages of oocytes at each stage are shown in Table 2. The average initial diameter for follicles that survived to day 10 (as defined above) was 367.9 ± 10.1 μm, while their day 10 average diameter was 409.4 ± 11.1 μm (mean ± SEM).

## 4. Discussion

In vitro culture conditions have been shown to influence the survival and development of isolated ovarian follicles in many species [3]. In the present study, we investigated the impact of ovarian stromal cells and conditioned medium supplemented at varying concentrations on the survival and growth of isolated preantral, early antral, and antral stage cat follicles. We found that (1) 100% conditioned medium from ovarian stromal cells best supported follicular survival in a 13-day culture period; (2) the initial developmental stage of follicles affected survival outcomes; (3) ovarian stromal cell and conditioned medium supplementation did not improve follicle growth or oocyte maturation rates; and (4) *CYP19A* expression was upregulated in the 50% CM treatment.

Ovarian stroma cells produce factors that regulate folliculogenesis, including VEGF, FGF2, IGF-1, and EGF [10]. These factors likely contributed to improved follicle survival in the 100% CM, as observed in the present study. However, we did not observe beneficial effects of co-culture treatment in supporting follicular survival. We hypothesized that over-confluency of stromal cells may have contributed to this finding. It is known that extended intervals between passages affect cell growth, differentiation, and necrosis rates, and high passage numbers can alter secretome expression and affect conditioned media composition [31,32]. In a study by Grubliauskaitė et al. [10], it has been shown that ovarian stromal cells support the growth of preantral human follicles for seven days. In the present study, cells were allowed to grow for 14 days (one day to adhere and 13 days of follicle culture). As the culture progressed, the cells visibly and consistently changed morphology, becoming irregular and elongated, with cell aggregation as they competed for space. Furthermore, the proportion of non-viable cells appeared to increase as cell culture progressed, especially on days 11 and 13 (Supplement Figure S1, Supplement Table S1). Morphologically, the cells also shifted from a mixture of elongated, fibroblast-like and cobblestone-like cells, to only long, thin fibroblast-like cells. Based on visual assessments, the morphology changes and confluency occurred on average by day 8. This is consistent with the previous study that assessed the time when cells achieved confluency [10], and further corresponds to a drop in co-culture follicle survival as compared to the 100% CM. It is likely that these stressed cells were secreting necrotic factors, such as death ligands, that are members of the tumor necrosis factor family, or concentrations of dissolved gases, such as nitric oxide, into the follicular culture [33]. Nitric oxide has been previously shown to negatively affect nearby cells in an overgrown co-culture schematic [34]. With increasing necrotic factors in the follicle culture, follicles likely could not survive, despite potential growth factors added by healthy cells. Future studies to assess nitric oxide production, necrotic factors, and reactive oxygen species will give valuable insights into how to improve the co-culture system.

Findings obtained from previous studies suggest that excess nutrients, especially carbohydrates, could cause metabolic stress in follicles, oocytes, and embryos, which in turn compromise development [35,36,37]. For example, a 50% reduction of carbohydrates results in normal development of mouse embryos at a comparable rate to the control [38], while cleaved bovine embryos were more likely to reach the blastocyst stage [39]. Because cultured cells consume glucose at a high rate, perhaps CM had a lower glucose concentration than the EGM (-) used in other cultured groups [40]. However, tested CM glucose concentrations averaged 5.5 ± 0.24 mmol/L, and there was no significant difference between CM and EGM (-) (Supplement Table S2). Therefore, it is unlikely that lowered glucose concentration played a role in the 100% CM follicle survival.

In the present study, follicle growth was unaffected by treatment, which is contrary to previous studies in which a growth benefit of CM or co-culture was demonstrated [10,41]. Differences in species, cell types, culture periods, and follicle stages may have contributed to variations among studies. One aspect that likely limited growth in this study was the concentration of alginate beads. While the alginate does provide needed structure to the follicle, it also restricts growth in antral stage follicles [42]. Alginate does not naturally biodegrade; therefore, the follicle cannot remodel the alginate as it would the cortex in vivo. To allow antral expansion, a fibrin–alginate interpenetrating network has been used [43]. Fibrin can be degraded by follicular enzymes and therefore provides room for follicles to expand as they grow [43].

In the present study, initial size had an impact on growth on days 4 and 13. The difference was between preantral and early antral follicles, with the former displaying higher growth on day 4, while the latter was higher on day 13. It is possible that preantral follicles experienced high proliferation by day 4, which led to their higher median growth rate. However, since 60% of preantral follicles that survived to day 13 did not grow an antrum, their growth rate dropped in comparison to early antral follicles, which can attribute their growth to antral expansion.

Oocyte maturation did not reveal any significant differences between treatment groups, nor did it yield a metaphase II oocyte (Table 2). Historically, in vitro maturation rates of cat oocytes have been approximately 50% [44]. It is important to note that, in this study, oocytes were collected from follicles and subjected to IVM regardless of survival status. This may have increased the relative numbers of apoptotic oocytes from follicles that had constricted growth. Also, follicles that survived to day 10 were, on average, 409 μm in diameter. Previous studies have shown that the percentage of morphologically normal oocytes that can be recovered from 400–800 μm feline follicles is around 20%, depending on the estrous cycle stage [45]. After in vitro maturation of those morphologically normal oocytes, 28% to 38% were degenerated, 37% to 54% were classified as GV, and 10% to 23% reached metaphase II [45]. Therefore, it is not surprising that no oocyte reached metaphase II in this study. The follicles were likely not mature enough to produce a developmentally competent oocyte. Furthermore, the culture period may have contributed to lowering mature oocyte numbers. Obtaining a follicle that can produce a developmentally competent oocyte is therefore a delicate balance between culture length, initial follicle size, and potential growth. If a follicle is too large initially, then it will likely degenerate before the end of culture (13 days), as evidenced by antral follicle survival rates in this study (Figure 2). Yet, a follicle that is too small may not grow enough during the culture period, and the chances of survival decrease the longer the culture continues. Solving this dilemma may include designing a multistep culture system or decreasing the alginate concentration to allow follicle expansion as in vitro culture progresses.

qRT-PCR analysis showed an upregulation in *CYP19A* expression in the 50% CM follicles. CYP19A, or aromatase, is the final enzymatic step for the production of estrogen [46]. This finding was unexpected, as the 50% CM improved neither follicle survival nor growth. Variations between follicle sizes in each qRT-PCR replicate may have contributed to this finding, although it is unlikely to be the complete explanation of why *CYP19A* was upregulated. Furthermore, upregulation of mRNA expression does not necessarily correlate with increased translation and protein production. In vitro-produced goat embryos showed an upregulation in *OCT4* mRNA expression but a decreased OCT4 protein expression [47]. Perhaps the 50% CM culture induced a similar phenomenon in the follicles, leading to higher mRNA expressions without producing a downstream effect on growth or survival. More research is required to elucidate these results, including measuring follicle steroid production in vitro.

## 5. Conclusions

The findings from our study indicate that ovarian stroma cell-conditioned media have a positive impact on the survival of cat pre- and early antral follicles. This finding serves as a foundation for future studies aimed at exploring survival factors secreted by ovarian stromal cells, vital information for developing an ideal in vitro microenvironment for cat folliculogenesis.

## Figures and Tables

**Table 1:** qRT-PCR primer information.

Gene ID	Forward Sequence (5′ to 3′)	Melting Temperature	Reverse Sequence (5′ to 3′)	Product Length (nt)	Accession Number	Source
*ACTG*	ATC CAC GAG ACC ACC TTC	54 °C	CAC CGT GTT AGC GTA GAG	75	XM_023244304.2	This study
*FSHR*	GGA TCT TTG CTT TCA TGG TC	51.6 °C	AAC ATA GAG CTG TGA CAA GG	113	NM_001048014.1	This study
*GDF9*	CAT CCG TGG ACC TGC TAT TT	54.8 °C	CCA GGT TGC ACA CAC ATT TC	129	NM_001165900.1	[23]
*CYP19A*	CAA TCC TGC TGC TCA CTG	53.6 °C	CCA TGC AAT AGC CAG GAC	84	GU306147.1	[24]

**Table 2:** Percentages of germinal vesicle (GV), germinal vesicle breakdown (GVBD), metaphase II (MII), or degenerated (D) oocytes recovered from control, co-culture, and 100% CM follicles and subjected to in vitro maturation for 24 h.

Treatment	Total Oocytes	GV (%)	GVBD (%)	MII (%)	D (%)
Control	43	9.3	46.5	0	41.9
Co-culture	46	6.5	47.8	0	39.1
100% CM	43	14	53.5	0	25.6

## Data Availability

The raw data supporting the conclusions of this article will be made available by the authors on request.

## Supplementary Materials

The following supporting information can be downloaded at https://www.mdpi.com/article/10.3390/ani15111539/s1, Figure S1: Live/dead staining of sham co-culture cells (Material and methods, Results); Table S1: The average percentage of dead cells in the sham co-culture at each experimental timepoint; Table S2: The average of corrected concentrations from ECM (⁻) and 16 aliquots of CM that were allowed to equilibrate to room temperature for 2 h.
