# Supplementary material for: Ovarian Stromal Cell-Conditioned Media, but Not Co-Culture, Improves Survival in Feline Follicles
Source: Animals (Basel). 2025 May 24;15(11):1539. doi: 10.3390/ani15111539 (PMC12153732; doi:10.3390/ani15111539)
Supplement: Supplementary file 1 [file animals-15-01539-s001.zip › animals-3553907-supplementary.pdf]

Supplement

Live/dead staining of sham co-culture cells (Materials and Methods, Results)

Six wells of ovarian stromal cells were seeded, cultured as described in the Materials and Methods. However, no ovarian follicle was added to the culture. This was referred to as a sham co-culture. At each experimental timepoint, one sham co-culture well was chosen for live/dead imaging. Cells were stained with ReadyProbe™ Cell Viability Imaging Kit, Blue/Green as per manufacturer recommendation (Invitrogen, R37609). After a five-minute incubation at 37°C, cells were imaged under a DAPI filter (all cells) and a GFP filter (dead cells) on an EVOS microscope. Five fields were captured in each well, then the well was discontinued from culture. This was repeated for two cultures (Supplementary Figure 1).

All stained cells were subsequently counted. The average number dead of cells per day was divided by the average number of total cells for that day to obtain average percent dead cells (Supplementary Table 1).

Average dead cells ranged from 3.34% on Day 0 to 13.57% on Day 13. However, it should be noted that this count only includes cells that were adhered to the culture well and had an intact nucleus. This count excludes any detached, apoptotic cells and cellular debris floating in the well.

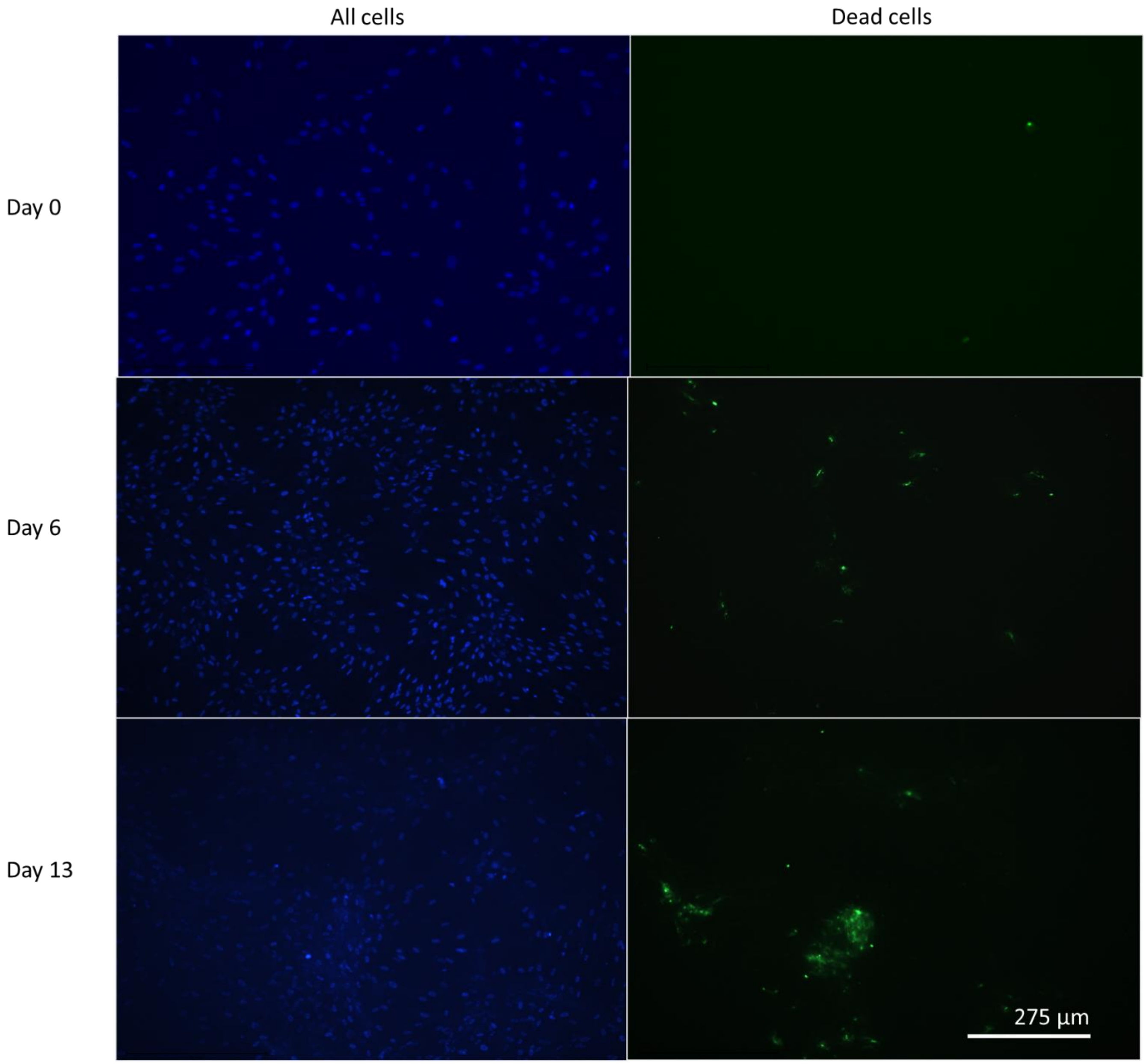

Supplementary Figure S1. Live/dead staining analysis of sham co-culture cells on Days 0, 6, and 13. (Left) All cells stained in blue. (Right) Dead cells stained in green. Scale bar is 275 µm.

Timepoint	Percent dead cells (Mean ± SD)
Day 0	3.3±2.6
Day 4	2.5±1.9
Day 6	2.5±0.8
Day 8	3.0±0.9
Day 11	6.6±4.4
Day 13	13.6±3.9

Supplementary Table S1. The average percentage of dead cells in the sham co-culture at each experimental timepoint.

Aliquot	Corrected Concentration Average (mmol/L)
EGM (-)	5.2
CM 1	4.35
CM 2	6.25
CM 3	3.15
CM 4	5.2
CM 5	6.2
CM 6	4.45
CM 7	6.4
CM 8	6.8
CM 9	6.7
CM 10	6.2
CM 11	5.6
CM 12	6.0
CM 13	5.9
CM 14	5.6
CM 15	5.1
CM 16	5.1

Supplementary Table S2. The average of corrected concentrations from EGM (-) and 16 aliquots of CM that were allowed to equilibrate to room temperature for 2 hours. Table S2: The average of corrected concentrations from ECM ($^-$) and 16 aliquots of CM that were allowed to equilibrate to room temperature for 2 h.
